# Shifts in the Gut Metabolome and *Clostridium difficile* Transcriptome throughout Colonization and Infection in a Mouse Model

**DOI:** 10.1128/mSphere.00089-18

**Published:** 2018-03-28

**Authors:** Joshua R. Fletcher, Samantha Erwin, Cristina Lanzas, Casey M. Theriot

**Affiliations:** a Department of Population Health and Pathobiology, College of Veterinary Medicine, North Carolina State University, Raleigh, North Carolina, USA; University of Kentucky

**Keywords:** *Clostridium difficile*, amino acids, intestinal colonization, metabolomics, peptides, transcriptomics

## Abstract

Antibiotics alter the gut microbiota and decrease resistance to Clostridium difficile colonization; however, the mechanisms driving colonization resistance are not well understood. Loss of resistance to C. difficile colonization due to antibiotic treatment is associated with alterations in the gut metabolome, specifically, with increases in levels of nutrients that C. difficile can utilize for growth *in vitro*. To define the nutrients that C. difficile requires for colonization and pathogenesis *in vivo*, we used a combination of mass spectrometry and RNA sequencing (RNA Seq) to model the gut metabolome and C. difficile transcriptome throughout an acute infection in a mouse model at the following time points: 0, 12, 24, and 30 h. We also performed multivariate-based integration of the omics data to define the signatures that were most important throughout colonization and infection. Here we show that amino acids, in particular, proline and branched-chain amino acids, and carbohydrates decrease in abundance over time in the mouse cecum and that C. difficile gene expression is consistent with their utilization *in vivo*. This was also reinforced by the multivariate-based integration of the omics data where we were able to discriminate the metabolites and transcripts that support C. difficile physiology between the different time points throughout colonization and infection. This report illustrates how important the availability of amino acids and other nutrients is for the initial stages of C. difficile colonization and progression of disease. Future studies identifying the source of the nutrients and engineering bacteria capable of outcompeting C. difficile in the gut will be important for developing new targeted bacterial therapeutics.

**IMPORTANCE** Clostridium difficile is a bacterial pathogen of global significance that is a major cause of antibiotic-associated diarrhea. Antibiotics deplete the indigenous gut microbiota and change the metabolic environment in the gut to one favoring C. difficile growth. Here we used metabolomics and transcriptomics to define the gut environment after antibiotics and during the initial stages of C. difficile colonization and infection. We show that amino acids, in particular, proline and branched-chain amino acids, and carbohydrates decrease in abundance over time and that C. difficile gene expression is consistent with their utilization by the bacterium *in vivo*. We employed an integrated approach to analyze the metabolome and transcriptome to identify associations between metabolites and transcripts. This highlighted the importance of key nutrients in the early stages of colonization, and the data provide a rationale for the development of therapies based on the use of bacteria that specifically compete for nutrients that are essential for C. difficile colonization and disease.

## Abstract (precis)

Clostridium difficile is a bacterial pathogen of global significance that is a major cause of antibiotic-associated diarrhea. Antibiotics deplete the indigenous gut microbiota and change the metabolic environment in the gut to one favoring C. difficile growth. Here we used metabolomics and transcriptomics to define the gut environment after antibiotics and during the initial stages of C. difficile colonization and infection. We show that amino acids, in particular, proline and branched-chain amino acids, and carbohydrates decrease in abundance over time and that C. difficile gene expression is consistent with their utilization by the bacterium *in vivo*. We employed an integrated approach to analyze the metabolome and transcriptome to identify associations between metabolites and transcripts. This highlighted the importance of key nutrients in the early stages of colonization, and the data provide a rationale for the development of therapies based on the use of bacteria that specifically compete for nutrients that are essential for C. difficile colonization and disease.

## INTRODUCTION

Clostridium difficile is a spore-forming, toxin-producing Gram-positive bacterial pathogen and is a major cause of antibiotic-associated diarrhea (1, 2). C. difficile infection (CDI) in humans has a range of clinical disease manifestations, including mild to severe diarrhea, pseudomembranous colitis, and the potentially lethal toxic megacolon (3). While antibiotic treatment can resolve CDI, as many as one-third of patients experience disease relapse, often multiple times (4). Nearly 500,000 infections and 29,000 deaths were directly or indirectly attributable to CDI in the United States in 2011, making it a significant source of morbidity and mortality in that country (5).

Antibiotic use is the most significant predisposing factor for susceptibility to CDI, as antibiotics alter the indigenous gut microbiota (6–9). The loss of microbially diverse populations, including key taxa from the *Lachnospiraceae* and *Ruminococcaceae* families, leads to a decrease in colonization resistance, which is the ability of an intact indigenous gut microbiota to prevent colonization by pathogens (10–12). This loss significantly alters the metabolic environment of the gut, changing the composition and concentration of bacterial and host-derived metabolites. One key metabolic pathway that is depleted is the conversion of host-derived primary bile acids to secondary bile acids by members of the gut microbiota (9, 12–14). C. difficile vegetative cell growth is significantly inhibited in the presence of secondary bile acids *in vitro*, and restoration of secondary bile acid metabolism in the gut is associated with an increase in resistance to C. difficile colonization (14–16).

While secondary bile acid metabolism could be a contributing factor in providing resistance to C. difficile colonization, antibiotic depletion of the gut microbiota also decreases abundances of members of the community that may have nutritional requirements similar to those of C. difficile, such as commensal species of Clostridia. This view is supported by evidence that precolonization of a susceptible host by nontoxigenic C. difficile can prevent toxigenic C. difficile colonization (17, 18). Early studies showed that cecal microbiota grown in germfree fecal pellet homogenates reduced C. difficile growth; this competition reverted when the homogenate was supplemented with glucose, N-acetylglucosamine, or N-acetylneuraminic acid (19). This suggests that an intact gut microbiota can consume or render inaccessible key nutrients that C. difficile requires for colonization.

Evidence from mouse models suggests that while C. difficile can adapt its metabolism to colonize susceptible hosts that have different gut microbial community structures (or none, in the case of germfree mice), there exist a subset of metabolic pathways that C. difficile utilizes irrespective of its environment *in vivo* (20–22), specifically, pathways for carbohydrate and amino acid fermentation, including amino acids for which C. difficile is an auxotroph. Our group has previously shown that susceptibility to C. difficile colonization is associated with antibiotic-induced shifts in the murine gut microbiome and, more importantly, in the postantibiotic metabolome, which was enriched in carbohydrates, sugar alcohols, peptides, and amino acids (9). This led us to hypothesize that the availability of specific nutrients that support C. difficile growth in the gut after antibiotic treatment is responsible for the observed decrease in colonization resistance and that colonization resistance in the gastrointestinal (GI) tract is, in part, mediated by competition for these specific nutrients by the members of an intact microbiota (23).

To understand how the gut microbiota is able to compete against C. difficile for similar nutrients, we first need to define the changes in gut nutrient levels that occur after treatment with antibiotics and, more importantly, as C. difficile colonizes a host. Availability of nutrients is also important for C. difficile pathogenesis and disease progression, as C. difficile virulence factor gene expression is exquisitely sensitive to nutrient availability (24, 25). Previous studies have explored C. difficile nutrient utilization *in vitro* in a defined medium over time; however, fewer studies have defined this in the more complex environment of the antibiotic-depleted host gut *in vivo* (24, 26–30). Other C. difficile studies addressing changes in the global gut metabolome at time points postantibiotic treatment *in vivo* have compared either data from one time point between multiple antibiotics or data from one antibiotic across long time scales, i.e., multiple weeks (9, 20).

To define the nutrients that C. difficile requires for colonization and pathogenesis *in vivo*, we used a combination of mass spectrometry and RNA sequencing (RNA Seq) to model the gut metabolome and C. difficile transcriptome throughout an acute infection in a well-characterized mouse model of CDI. We also performed multivariate-based integration of the gut metabolome and C. difficile transcriptome to define the signatures that were the most important throughout infection at each of the time points after challenge with C. difficile spores.

Here we used a two-tiered approach combining metabolomics with transcriptomics *in vivo* and found that C. difficile uses specific amino acids and carbohydrates early in the process of colonization of a susceptible host. This finding was also reinforced by the multivariate-based integration of the omics data. We were able to discriminate the metabolites and transcripts that support C. difficile physiology between the different time points throughout colonization and infection. This report illustrates how important the availability of amino acids and other nutrients is for the initial stages of C. difficile colonization and progression of disease. Future studies identifying the source of the nutrients and engineering bacteria capable of outcompeting C. difficile in the gut will be important for developing new targeted bacterial therapeutics.

## RESULTS

### C. difficile colonization and infection are associated with significant changes in the cecal metabolome.

Mice sacrificed at 12, 24, and 30 h postchallenge with C. difficile were all colonized and had averages of 1.13 × 10^6^ CFU/g cecal content at 12 h, 1 × 10^7^ CFU/g cecal content at 24 h, and 4.75 × 10^8^ CFU/g cecal content at 30 h. Clinical signs of disease were monitored and peaked at 30 h after challenge.

**FIG 1:**
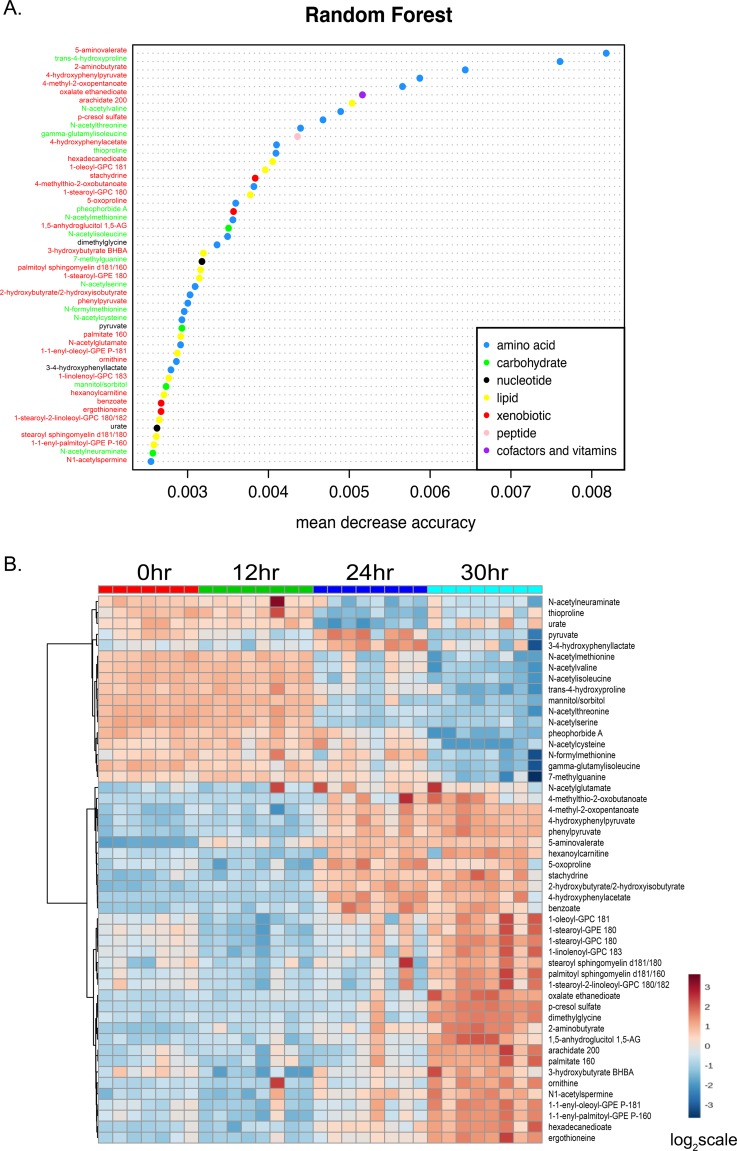
Cecal metabolome during C. difficile colonization and infection. (A) Variable-importance plot of the top 50 metabolites identified by Random Forest analysis. The mean accuracy value decrease is a measure of how much predictive power is lost if a given metabolite is removed or permuted in the Random Forest algorithm; thus, the more important a metabolite is to classifying samples into time point categories, the further to the right its point is on the graph. Metabolite points are color-coded according to the KEGG superpathway in which they belong. Metabolite names are labeled red if their level increased throughout infection, black if they were variable, and green if the level decreased. (B) Heat map showing the relative abundances of the metabolites identified in panel A. Each column corresponds to the cecal metabolome from an individual mouse, and each row corresponds to a given metabolite. Unsupervised hierarchical clustering was used to cluster metabolites with similar abundance profiles over time. The heat map scale ranges from −3 to 3 on a log_2_ scale.

To characterize the temporal changes in the nutritional environment during C. difficile colonization and disease, untargeted metabolomics was performed on cecal content harvested from mice at 0, 12, 24, and 30 h postchallenge with C. difficile VPI 10463 in a cefoperazone-induced mouse model of CDI. Analysis of variance (ANOVA) identified 482 metabolites that differed significantly between time points, with most changes in abundance occurring by 24 and 30 h postchallenge relative to time zero (one-way ANOVA) (see Table S2 in the supplemental material). Random Forest analysis was applied to identify metabolites that are important for distinguishing time points (31) (Fig. 1A). It is a classification method that analyzes multiple variables (metabolites) between different groups of samples (time points) and uses variability between the groups to classify a given sample (murine cecal metabolome) as belonging to a given group. It identifies the variables that contribute most to the classification of a sample to a group.

The mean decrease in accuracy (MDA) score data depicted in Fig. 1A represent the predictive accuracy that a given metabolite has for assigning a sample to a time point. Metabolites that significantly change in abundance over time are more likely to be identified as important for classifying samples to time points, as data corresponding to a metabolite with values that do not change look similar across all time points. Metabolites that increased in abundance over time are labeled in red font, those that decreased in abundance over time in green, and those that were variable in abundance in black (Fig. 1A).

Twenty-four of the top 50 metabolites with the highest MDA score belonged to the amino acid Kyoto encyclopedia of genes and genomes (KEGG) metabolic pathway, consistent with C. difficile nutrient utilization *in vitro* (Fig. 1A). 5-Aminovalerate had the highest MDA score, and the value increased during infection. 5-Aminovalerate is the by-product of the fermentation of proline via Stickland metabolism. This is a reaction that is unique to some members of the Clostridia, including C. difficile (32). The metabolite with the second-highest MDA score from the Random Forest analysis was *trans*-4-hydroxyproline, a posttranslationally modified form of proline that is often found in collagen; its score decreased over time, which is consistent with the production of 5-aminovalerate (33). Fourteen of the top 50 metabolites identified were lipid species, many of which were phospholipids likely derived from host cell membranes. Two non-cell-membrane-associated lipids, 3-hydroxybutyrate and hexanoylcarnitine, were also identified. Of the four carbohydrates, two, namely, mannitol/sorbitol and the sialic acid 5-acetylneuraminate, have previously been connected to C. difficile colonization and metabolism (9, 19). Pyruvate, an intermediate in multiple metabolic pathways, was also identified as being significant for differentiating samples over time.

### C. difficile colonization and infection are associated with decreased levels of N-acetylated amino acids, carbohydrates, and sugar alcohols and increased levels of metabolic by-products and lipids.

We next generated an unsupervised heat map to visualize the changes in the relative abundances of the top 50 metabolites identified via Random Forest analysis throughout infection (Fig. 1B). Nine metabolites (N-acetylneuraminate, thioproline, N-acetylmethionine, N-acetylvaline, N-acetylisoleucine, *trans*-4-hydroxyproline, mannitol/sorbitol, N-acetylthreonine, and N-acetylserine) had similarly high relative abundances at the 0 h and 12 h time points; however, all had decreased in abundance by 24 h, and the levels remained low at 30 h postchallenge with C. difficile (Fig. 1B).

A second set of 12 metabolites exhibited the opposite pattern, where the relative abundances were low at the 0 h and 12 h time points but were increased by 24 and 30 h postchallenge with C. difficile (Fig. 1B). These included N-acetylglutamate, which is produced during conversion of glutamate to ornithine; 4-methylthio-2-oxobutanoate, a by-product of methionine metabolism that also contributes to methionine salvage; 4-methyl-2-oxopentanoate, a by-product of leucine fermentation; 4-hydroxyphenylpyruvate, an intermediate in the C. difficile-specific tyrosine–to–p-cresol pathway; phenylpyruvate, derived from the oxidative deamination of phenylalanine; 5-aminovalerate, the by-product of proline fermentation; hexanoylcarnitine, an acylcarnitine possibly associated with mitochondrial beta-oxidation; 5-oxoproline, an intermediate in the gamma-glutamyl cycle of eukaryotes; stachydrine (also known as proline betaine), an osmoprotectant that some bacteria can utilize as a carbon and nitrogen source; 2-hydroxybutyrate, derived from threonine and methionine metabolism; 4-hydroxyphenylacetate, another intermediate in the formation of p-cresol; and benzoate, a carboxylic acid that some anaerobic methanogens can produce from phenol (34–44).

Five metabolites identified in the Random Forest analysis had high relative abundances until the 30 h time point, suggesting that there may be a hierarchy of consumption of nutrients in the antibiotic-depleted gut environment. Pyruvate, pheophorbide A, N-acetylcysteine, N-formylmethionine, gamma-glutamyl-isoleucine, and 7-methylguanine were decreased in abundance at 30 h (Fig. 1B), suggesting a switch to pyruvate fermentation and alternative amino acid sources for further amino acid fermentation. Pheophorbide A is a degradation product of chlorophyll (45), though its provenance was unclear in our model. 7-Methylguanine is a purine associated with RNA 5′ capping and DNA alkylation, and while C. difficile has numerous enzymes for purine metabolism, it is unclear if it can be utilized for growth. The amino acids and carbohydrates that were depleted by 24 h largely remained low in abundance at 30 h. The fatty acid end products of amino acid fermentation 2-aminobutyrate, caproate, isocaproate, and valerate were all of low relative abundance until 30 h postchallenge. The relative abundance of p-cresol, an end product of tyrosine fermentation, also increased at the 30 h time point. Additionally, we detected similar patterns of change in numerous carbohydrates, such as sucrose, mannitol/sorbitol, isomaltose, etc., which were abundant early but decreased in abundance by 24 h (see Fig. S1 in the supplemental material). Many more carbohydrates, including glucose and fructose, among others, remained abundant until 30 h. This represents evidence that numerous nutrients that C. difficile either requires or is known to utilize (24, 36, 46–48) had been consumed early during colonization.

10.1128/mSphere.00089-18.2 FIG S1  Heat maps of the relative abundances of carbohydrates and peptides not included in the Random Forest analysis described in the Fig. 1 legend. Each column corresponds to the cecal metabolome from an individual mouse, and each row corresponds to a given metabolite. Unsupervised hierarchical clustering was used to cluster metabolites with similar abundance profiles over time. The heat map scale ranges from −4 to 4 on a log_2_ scale. Download FIG S1, PDF file, 0.8 MB. Copyright © 2018 Fletcher et al. 2018 Fletcher et al. This content is distributed under the terms of the Creative Commons Attribution 4.0 International license.

In the ceca from six of eight mice at the 30 h time point, we detected increases in the abundance of 30 di- and tripeptides, including 11 gamma-glutamyl amino acids (Fig. S1). These species may have been generated by the activity of host extracellular gamma-glutamyl transferase, which transfers the gamma-glutamyl moiety of glutathione to acceptor molecules, including amino acids, for cellular uptake (49, 50). The remaining 19 dipeptides are heavily biased toward those containing leucine, valine, and glutamine (Fig. S1). Additionally, in the ceca from the same six of eight mice at the 30 h time point, we detected increases in the levels of the following 16 amino acids: histidine, methionine, tyrosine, phenylalanine, tryptophan, glutamine, serine, cystine, leucine, isoleucine, valine, arginine, taurine, ornithine, alanine, and lysine (Fig. S2). This may indicate the presence of proteases, either host or bacterial, that could have acted on proteins and liberated the peptides and amino acids into the cecal milieu.

10.1128/mSphere.00089-18.3 FIG S2  Heat maps of the relative abundances of amino acids and lipids not included in the Random Forest analysis described in the Fig. 1 legend. Each column corresponds to the cecal metabolome from an individual mouse, and each row corresponds to a given metabolite. Unsupervised hierarchical clustering was used to cluster metabolites with similar abundance profiles over time. The heat map scale ranges from −4 to 4 on a log_2_ scale. Download FIG S2, PDF file, 21.4 MB. Copyright © 2018 Fletcher et al. 2018 Fletcher et al. This content is distributed under the terms of the Creative Commons Attribution 4.0 International license.

The largest number of changes at 30 h postchallenge came from the KEGG superpathway for lipids, with a majority of the 237 lipid species identified via untargeted metabolomics showing relatively low abundance over 0 to 24 h but then increasing in abundance at 30 h postchallenge with C. difficile (Fig. S2). These included but were not limited to the following: short-, medium-, and long-chain fatty acids; phospholipids and glycerolipids; host-derived endocannabinoid species; inflammatory mediators; sphingolipids; and lysolipids.

### The C. difficile transcriptome changes significantly throughout colonization and infection.

**FIG 2:**
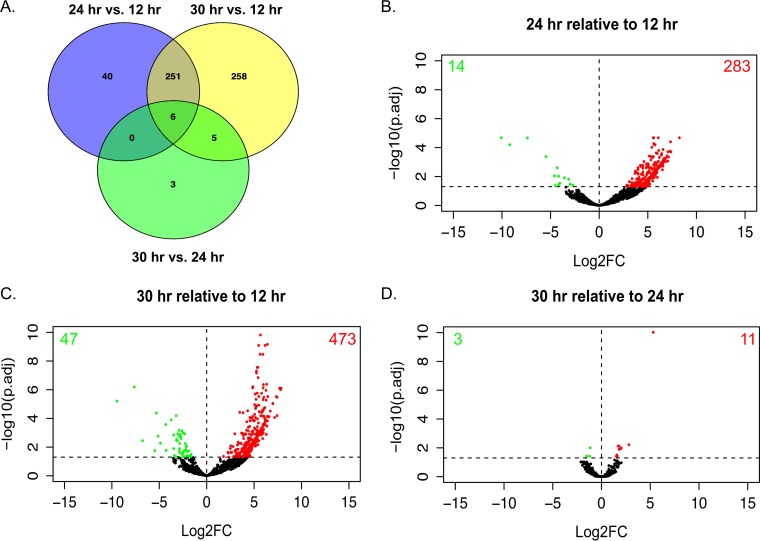
C. difficile transcriptome during colonization and infection. (A) Venn diagram showing the differentially expressed genes that were shared or unique between the three time points. (B to D) Volcano plots highlighting genes whose transcript levels changed by greater than 2-fold and met the significance threshold *P* adj. = <0.05. Genes highlighted in red had increased transcript levels, while those highlighted in green had decreased levels. Points in black represent genes whose results failed to meet the significance threshold.

We next performed RNA Seq analysis on paired cecal content samples from the 12, 24, and 30 h time points. This analysis also surveys changes in the metabolome that correspond to the metabolic capacity encoded in the C. difficile genome and its gene expression *in vivo*. Stranded, paired-end reads were mapped to the C. difficile VPI 10463 (ATCC 42355) genome. Differential expression analysis was used with DESeq2, comparing the time points throughout infection to each other for a total of three comparisons: 24 h versus 12 h, 30 h versus 12 h, and 30 h versus 24 h (Fig. 2; see also Table S3) (51, 52). At 24 h relative to 12 h, we detected 297 differentially expressed genes (DEGs), with 14 genes decreased in expression and 283 increased (Fig. 2A and B). Relative to 12 h, at the 30 h time point we detected 520 DEGs, with 47 genes decreased in expression and 473 increased (Fig. 2C). In the final comparison, 30 h relative to 24 h, we detected 14 DEGs, with 3 genes being decreased in expression and 11 increased (Fig. 2D). Accordingly, there was significant overlap of the DEGs at the 24 h and 30 h time points relative to 12 h (Fig. 2A). Of the 258 unique DEGs at 30 h relative to 12 h, many are also expressed at 24 h, though they failed to meet the adjusted *P* value cutoff. The expression patterns of select genes from all three comparisons were confirmed via quantitative reverse transcriptase PCR (Fig. S3).

10.1128/mSphere.00089-18.4 FIG S3  Quantitative reverse transcriptase PCR of six genes identified as differentially expressed in the RNA Seq analysis. Expression was quantified via a standard curve and was normalized to the expression of *rpoC*. Information on primers can be found in Table S1. Download FIG S3, PDF file, 0.8 MB. Copyright © 2018 Fletcher et al. 2018 Fletcher et al. This content is distributed under the terms of the Creative Commons Attribution 4.0 International license.

10.1128/mSphere.00089-18.6 TABLE S1  Primers used in quantitative reverse transcriptase PCR described in the Fig. S4 legend. Download TABLE S1, DOCX file, 0.1 MB. Copyright © 2018 Fletcher et al. 2018 Fletcher et al. This content is distributed under the terms of the Creative Commons Attribution 4.0 International license.

10.1128/mSphere.00089-18.7 TABLE S2  Significantly altered metabolite from each time point during colonization and infection. Each Excel tab corresponds to a metabolite statistical comparison between the groups listed done by a one-way ANOVA. The numbers of metabolites that were statistically significant in each comparison of data between groups are in red if they represent increases and in green if they represent decreases. Download TABLE S2, XLSX file, 0.2 MB. Copyright © 2018 Fletcher et al. 2018 Fletcher et al. This content is distributed under the terms of the Creative Commons Attribution 4.0 International license.

10.1128/mSphere.00089-18.8 TABLE S3  Results of the DESeq2 analysis. Download TABLE S3, XLSX file, 1.2 MB. Copyright © 2018 Fletcher et al. 2018 Fletcher et al. This content is distributed under the terms of the Creative Commons Attribution 4.0 International license.

Among the genes whose expression we detected as increased at 24 and 30 h were those whose encoded proteins are involved in the uptake and metabolism of carbohydrates, amino acids, and fatty acids, including those responsible for butyrate production (Table S3). The most highly induced gene at both 24 h and 30 h relative to 12 was a putative phage holin gene, with induction increases of 300- and 200-fold, respectively. This holin gene is within a genomic locus predicted to encode an incomplete prophage, so its role in C. difficile physiology *in vivo* is unclear. The second most highly induced gene at 30 h relative to 12 was *feoB*, encoding a ferrous iron importer. At 24 h, *feoB* induction was increased >30-fold but was increased >200-fold just 6 h later. This strongly implicates iron scarcity as a nutritional signal for C. difficile in the later stages of colonization, as *feoB* levels are inversely correlated with iron availability (53). Among the most highly induced genes at both 24 and 30 h were two copies of *brnQ*, encoding a branched-chain amino acid importer (54). The three genes whose induction was most highly increased at 30 h relative to 24 h were those encoding acetyl coenzyme A (acetyl-CoA) C-acetyltransferase, acetyl-CoA—acetoacetyl-CoA transferase subunit alpha, and 3-hydroxybutyrate dehydrogenase, involved in short-chain fatty acid metabolism and butyrate production. This is consistent with the increase in levels of short-chain fatty acids and the decrease in levels of pyruvate observed at 30 h in the metabolomics analysis, as pyruvate could serve as a precursor for increased acetyl-CoA production to fuel increased beta-oxidation of fatty acids.

### Global changes in C. difficile metabolic gene expression throughout colonization and infection.

**FIG 3:**
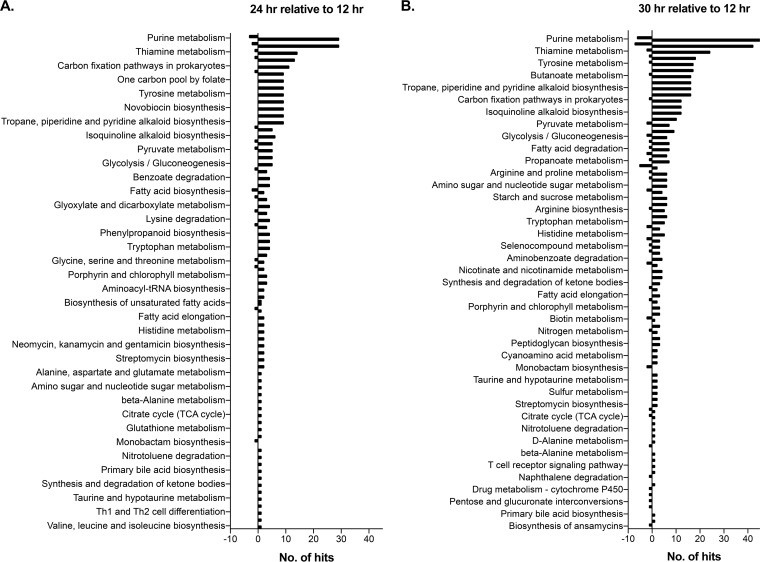
KEGG pathway analysis of the C. difficile DEGs throughout colonization and infection. Protein sequences of the DEGs at 24 h (A) or 30 h (B) relative to 12 h were imported into Blast2GO, and data corresponding to the predicted enzymes were loaded onto KEGG pathway maps. The numbers on the *x* axis correspond to the number of predicted enzymes used to map to a given pathway and whether the enzyme’s transcript was increased or decreased in expression.

The predicted protein-coding sequences for all DEGs were imported into Blast2GO for Gene Ontology annotation (Fig. S4). For the 24 h versus 12 h DEGs, 237 of 297 successfully completed the Blast2GO pipeline and were assigned Gene Ontology (GO) annotations, as were 390 of 520 DEGs from the 30 h versus 12 h comparison; 12 of 14 completed the pipeline from the 30 h versus 24 h comparison. Many of those that did not complete the pipeline were proteins of unknown function for which no GO annotation could be assigned or those with no homologs identified via BLAST. Enzyme Commission (EC) codes were assigned to all DEGs predicted to encode enzymes; these were then overlaid onto KEGG pathway maps (Fig. 3). This approach identifies all pathways onto which an EC code maps; thus, some enzymes encoded by our DEGs mapped to multiple pathways. Numerous enzymes mapped to KEGG pathways for the biosynthesis or degradation of several amino acids, including many identified as important in the metabolomics Random Forest analysis (Fig. 3). The category with the largest number of hits at both 24 and 30 h relative to 12 h includes enzymes predicted to function in purine metabolism, consistent with the need to replicate DNA and transcribe genes into RNA as the population of C. difficile rapidly increases *in vivo*. The KEGG pathway corresponding to biosynthesis of antibiotics was found to map a significant proportion of the predicted enzymes of DEGs at both 24 and 30 h. This category encompasses contributions from diverse pathways, including the following: glycolysis; the pentose-phosphate pathway; the shikimate pathway; the tricarboxylic acid (TCA) cycle; terpenoid biosynthesis; and purine, amino acid, amino sugar, and nucleotide metabolism. The number of DEGs that are predicted to be involved in oxidation-reduction reactions, as well as in transmembrane transport, and the number of DEGs that mapped to KEGG pathways for amino acid metabolism represent strong evidence that a significant portion of the C. difficile transcriptome *in vivo* is dedicated to their acquisition and metabolism. Indeed, network analysis of predicted protein-protein interactions via the STRING database identified statistically significant enrichment for genes in KEGG pathways involved in amino acid degradation and butyrate production, among others, among the genes with increased expression at 24 and 30 h; enrichment of pathways for glycolysis and fructose/mannose metabolism was observed in the transcripts that had decreased levels at 30 h relative to 12 h (Table S4).

10.1128/mSphere.00089-18.5 FIG S4  Blast2GO Gene Ontology annotations for the differentially expressed genes from each time point comparison during colonization and infection. Download FIG S4, PDF file, 0.8 MB. Copyright © 2018 Fletcher et al. 2018 Fletcher et al. This content is distributed under the terms of the Creative Commons Attribution 4.0 International license.

10.1128/mSphere.00089-18.9 TABLE S4  Results of the STRING 10.5 database KEGG pathway enrichment analysis. Each sheet in the Excel workbook corresponds to the enriched pathways that were decreased or increased in expression in each comparison. Among the genes that were decreased in expression in the comparisons of 24 h versus 12 h and then 30 h versus 24 h, no KEGG pathways were enriched; thus, they are not included in the Excel workbook. Download TABLE S4, XLSX file, 0.04 MB. Copyright © 2018 Fletcher et al. 2018 Fletcher et al. This content is distributed under the terms of the Creative Commons Attribution 4.0 International license.

**FIG 4:**
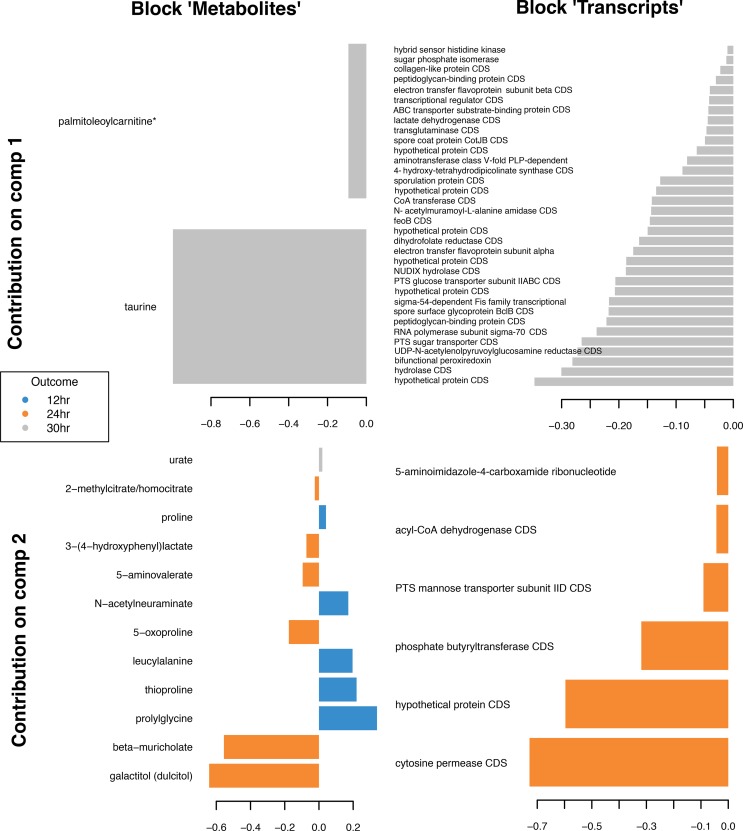
Multivariate-based analysis of the gut metabolome and C. difficile transcriptome during colonization and infection. A loading plot of the features selected in each component is provided. The top row indicates the features in the first component for the metabolites (left) and transcripts (right). The bottom row indicates the features in the second component for the metabolites (left) and transcripts (right). The values corresponding to the specific bar magnitudes are indicated in Table S5. The color indicates the expression levels of each variable according to each class where blue represents 12 h, orange represents 24 h, and gray represents 30 h.

**FIG 5:**
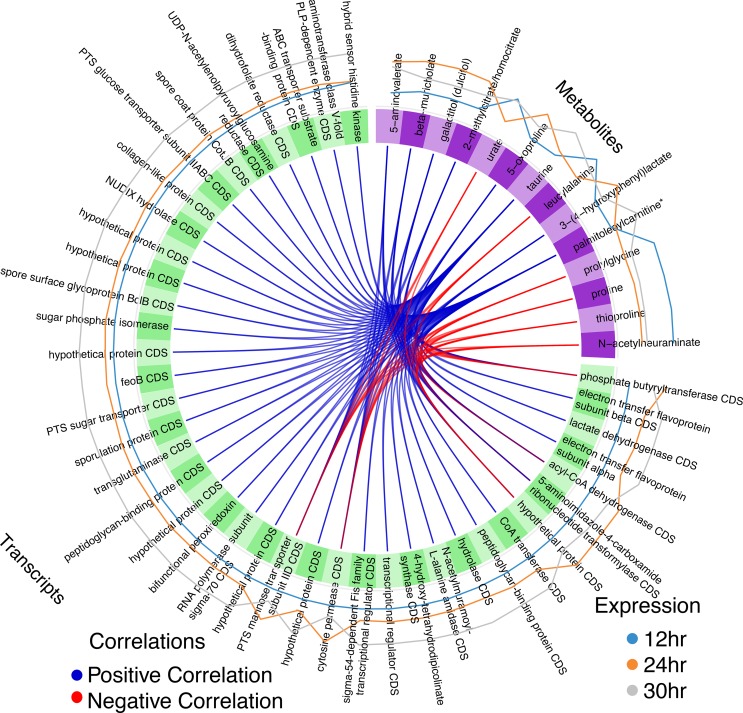
Plot of the correlations between the metabolome and C. difficile transcriptome. A Circos plot displays the positive and negative correlations (*r* = >0.7) between the selected features with blue and red lines, respectively. The values corresponding to the exact weight for each line are indicated in Table S5. The metabolites are indicated in purple (top right quadrant), and the transcripts are indicated in green. Each individual feature name is labeled in the block. The outer lines indicate the expression levels of each variable according to each class where blue represents 12 h, orange represents 24 h, and gray represents 30 h. CDS, coding sequence; PLP, proteolipid protein.

10.1128/mSphere.00089-18.10 TABLE S5  The numeric weights of the loading vectors indicated in Fig. 4. The color indicates the class in which the variable corresponds to the average level of expression as follows: blue represents 12 h, orange represents 24 h, and gray represents 30 h. The numeric weights of each line in the Circos plot are indicated in this table. Note that the Circos plot includes only those correlations between metabolites and transcripts whose values were greater than 0.7; as such, the correlations from metabolite to metabolites are not included in Fig. 5. Download TABLE S5, XLSX file, 0.1 MB. Copyright © 2018 Fletcher et al. 2018 Fletcher et al. This content is distributed under the terms of the Creative Commons Attribution 4.0 International license.

### Multivariate-based integration of the gut metabolome and C. difficile transcriptome throughout colonization and infection.

To identify associations between the gut metabolome and C. difficile transcriptome, we performed a sparse partial least-squares-discriminant analysis (sPLS-DA) utilizing the mixOmics package. The aim of the analysis was to identify a highly correlated multiomics signature discriminating the 12, 24, and 30 h time points throughout colonization and infection. Our final sPLS-DA model contained two components. The loading plots for the first component and second component are shown in Fig. 4 at the top and bottom, respectively (Table S5). Transcriptomic features dominated the first component. We found only two metabolites and 34 transcripts in the first component, all representing the 30 h time point (Fig. 4, top). This suggests that there were significant changes in the C. difficile transcriptome at the 30 h time point compared to the 12 h and 24 h time points. The second component was made up of 12 metabolites and six transcripts (Fig. 4, bottom). The metabolites in the second component, which were primarily amino acids, differed across all time points but predominantly represented changes in the early stages of colonization at the 12 h and 24 h time points as indicated by the color of each of the bars in Fig. 4.

Furthermore, we plotted the correlation between the selected metabolites and transcripts (*r* = >0.7). We observed strong correlations between each transcript and at least two metabolites, which further highlights the association of each feature with specific metabolites. Transcripts encoding 5-aminoimidazole-4-carboxamide ribonucleotide transformylase, acyl-CoA dehydrogenase, phosphotransferase system (PTS)-mannose transporter sub-IID, phosphate butyryltransferase, a hypothetical protein, and a cytosine permease had the most connections to metabolites. As shown in Fig. 5, all the aforementioned transcripts had positive correlations with 2-methylcitrate/homocitrate, 3-(4-hydroxyphenyl) lactate, 5-aminovalerate, 5-oxoproline, beta-muricholate, and galactitol (dulcitol). Likewise, these same transcripts all had negative correlations with leucylalanine, N-acetylneuraminate, palmitoleoylcarnitine, proline, prolylglycine, taurine, and thioproline. Additionally, urate was negatively correlated with a hypothetical protein, PTS-mannose transporter sub-IID, and cytosine permease. The data corresponding to the exact weight determined for each line are listed in Table S5.

## DISCUSSION

The rapid kinetics of the C. difficile VPI 10463 life cycle during colonization of a susceptible host have been well described; however, global temporal changes to the metabolome and C. difficile transcriptome during the early stages of colonization by this strain have not been examined (13). Although there are differences among the C. difficile strains used in various mouse models, as well as differences in the models, our metabolomic and transcriptomic results are in accordance with those of other *in vivo* studies that identified amino acid and carbohydrate metabolism as being important during C. difficile colonization and infection (20–22). Our results also further highlight the importance of the inverse relationship between the indigenous gut microbiota and nutrient levels, including many nutrients that are essential for C. difficile colonization. For example, due to auxotrophy and nutritional preference in complex media, proline has been identified as a nutrient of significance for C. difficile (9, 20, 55–57). Germfree mice have significantly higher levels of proline in their ceca, consistent with the increase observed in antibiotic-treated mice, suggesting that an intact microbiota is responsible for the low relative abundance of proline in mice that show resistance to colonization by C. difficile (58). In support of this, the top two metabolites identified by Random Forest analysis were 5-aminovalerate and *trans*-4-hydroxyproline. *trans*-4-Hydroxyproline decreased in relative abundance, while 5-aminovalerate increased, consistent with the utilization of the former. Indeed, hydroxyproline can substitute for proline *in vitro* and is a major constituent of collagen, one of the most abundant proteins in the body (55, 59). Recently, a glycyl radical enzyme from C. difficile has been shown to mediate dehydration of hydroxyproline, likely supplying the bacteria with a further source of proline (60, 61). In our study, the expression of *hypD*, the gene encoding the glycyl radical enzyme, was increased at 24 h but had decreased significantly by 30 h, suggesting that it may be responsive to hydroxyproline levels (see Fig. S3 in the supplemental material).

Random Forest analysis also identified several N-acetylated amino acids with high MDA scores. These were abundant early but had decreased in abundance by 24 h. N-acetylation is a common posttranslational modification in eukaryotes; therefore, some of the N-acetylated amino acids may have been derived from degradation of mouse proteins present in the gut (62). Many of these N-acetylated amino acids, as well as non-N-acetylated amino acids, including many which C. difficile has been demonstrated to use preferentially *in vitro*, were found to be relatively abundant early (57). Notably, valine and proline are essential amino acids for C. difficile, while the absence of methionine leads to extremely poor growth *in vitro*, suggesting that C. difficile may prioritize the acquisition and consumption of amino acids *in vivo* during colonization (26). Indeed, the non-N-acetylated forms of five of these amino acids make up over half of those required by C. difficile in minimal defined media (24), highlighting their importance.

Many of these amino acids are known to affect the life cycle of C. difficile. Threonine, when included in a cocktail of eight other amino acids, contributes to suppression of toxin synthesis (24). Isoleucine and valine are branched-chain amino acids that are known Stickland reaction donors, supplying reducing power to proline reductase through NADH and resulting in NAD+ and 5-aminovalerate (55, 56). The abundance of their N-acetylated forms had decreased by 24 h and remained low at 30 h. Given the increased expression of the *brnQ* genes encoding the branched-chain amino acid importer, it is likely that C. difficile imports significant amounts of these amino acids to supply Stickland donors for proline fermentation. The *codY* global transcriptional regulator gene in both C. difficile and Staphylococcus aureus regulates the *brnQ* genes, and *brnQ* has a demonstrated role in pathogenesis in the latter organism (63, 64). CodY is an allosteric regulator that mediates repression of most of its regulon when bound by branched-chain amino acids or GTP, including the toxin genes *tcdA* and *tcdB* (65). In addition to the amino acids identified via Random Forest analysis, the abundance of the carbohydrates N-acetylneuraminate and mannitol/sorbitol was observed to be high early but decreased by 24 h. They have been linked to C. difficile colonization of susceptible hosts, and C. difficile can utilize them for growth *in vitro* (9, 20) Consistent with this is the observation that several of the C. difficile genes encoding carbohydrate uptake systems, for example, those encoding PTS transporters, are increased in expression by 24 h. Also increased in expression were the genes of the *had* operon, responsible for leucine fermentation. The *had* genes were previously reported to be negatively regulated by the global carbon catabolite repressor CcpA in C. difficile strain JIR8094 (66). Similarly to CodY, CcpA is a negative repressor of toxin gene expression, though its activity is responsive to carbohydrates rather than to branched-chain amino acids/GTP. Therefore, the derepression by 24 h of the known CodY and CcpA targets *brnQ*, the *had* operon, *tcdA*, and *tcdB*, as well as many others, is strong evidence that C. difficile had depleted the local pools of these key nutrients and was experiencing nutrient starvation.

C. difficile is proteolytic, with several proteases and peptidases known to be involved in various processes, including cell adhesion, motility, biofilm, and germination (67–74). The expression of several proteases and peptidases was increased at 24 h in the C. difficile transcriptome and even more at 30 h (Table 1). Some of these were likely housekeeping proteases, such as Clp, which would be predicted to increase in expression, as the rapidly growing population of C. difficile cells would encounter cellular stressors in the form of antimicrobial peptides or other host defense mechanisms, especially after toxin-induced inflammation. Others may play a role in nutrient acquisition in the host, as evidenced by the increase in expression of numerous free amino acids and dipeptides at 30 h. Two such genes are predicted to encode β-aspartyl-peptidases that are components of the glycine reductase complex. Glycine is another amino acid that is fermented via the Stickland reaction, and nine of the peptides found to be increased in most mice at 30 h contained glycine. The remaining genes encoding the glycine reductase were significantly increased in expression at 24 and 30 h. Another gene encodes a product that is predicted to be a member of the S41 family peptidase. Its predicted protein is homologous to CtpA, a protease linked to pathogenesis in multiple Gram-negative pathogens and S. aureus, though it is unclear if those homologs are active on host proteins (75–78). Regardless, these proteases and peptidases remain targets for future investigation into the molecular pathogenesis of C. difficile* in vivo*.

**TABLE 1:** Proteases and peptidases that were differentially expressed throughout C. difficile colonization and infection^a^

Protease	Protein ID	Log_2_ fold change	
		24 h	30 h
Clp protease	WP_003428224.1	5.71**	5.17**
Aminopeptidase	WP_009902261.1	5.92**	5.50***
Peptidase S41	WP_003437815.1	5.05**	4.87**
Beta-aspartyl-peptidase	WP_003416871.1	4.59****	4.33****
Beta-aspartyl-peptidase	WP_004454406.1		4.96**
Serine protease	WP_011861421.1		4.40*
d-Alanyl-d-alanine-carboxypeptidase	WP_003428267.1		4.33*
Peptidase	WP_011862025.1		4.21*
Zinc metallopeptidase	WP_003416253.1		−3.48**

a ID, identifier. *, *P* adj. of <0.05; **, *P* adj. of <0.01; ***, *P* adj. of <0.001; ****, *P* adj. of <0.0001.

The largest class of metabolites for which we detected changes throughout infection was that of the lipids, where expression of a majority was significantly increased by 30 h postchallenge. As the activity of the C. difficile toxins TcdA and TcdB was evident by 30 h and as several of these lipid species are derived from the host, we interpret this to mean that the extensive cellular and tissue damage present at that time point had led to an influx of cellular debris and lipid signaling species into the lumen of the cecum. Indeed, numerous endocannabinoid species were detected, as was the inflammatory mediator 12-HETE, consistent with the highly inflammatory nature of the host cell response to intoxication by TcdA and TcdB activity (79).

Finally, our study had several limitations, the most important of which was the inability to confirm that the detected changes in the cecal metabolome were due specifically to the metabolism of C. difficile. Previous work by our group showed that members of the *Lactobacillus* genus remain in the cecum after antibiotic treatment (9, 13, 80). Therefore, while most of the changes in the cecal metabolome are likely due to the presence of C. difficile, given the limited taxonomical distribution of certain metabolic pathways, e.g., Stickland metabolism, it can be assumed that the host and the remaining microbiota could contribute to changes in the metabolome that occur either directly or indirectly in response to the presence of C. difficile or independently of it. While steps were taken to increase specificity in the RNA Seq analysis, we cannot rule out the possibility that some of the reads that mapped to the C. difficile genome were derived from the transcripts of highly conserved genes present and expressed in other species in the murine ceca from which the RNA was isolated. Additionally, our study examined the metabolome and C. difficile transcriptome only in mice treated with cefoperazone. Antibiotics with different mechanisms of action would target different classes of bacteria, leading to dissimilar community structures and thus to dissimilar metabolic environments. Jenior et al. found that to be the case in mice pretreated with different antibiotics; they observed that C. difficile adapted its gene expression to each environment at 18 h postchallenge with C. difficile (20).

The two-tiered approach of combining metabolomics with transcriptomics *in vivo* reinforced the idea that C. difficile uses certain amino acids and carbohydrates early in the process of colonization of a susceptible host. This was supported by the multivariate-based integration of the omics data. We could discriminate the metabolites and transcripts required for C. difficile physiology by different time points throughout infection. In particular, the abundance of proline-containing peptides and the N-acetylated forms of methionine, threonine, and branched-chain amino acids decreased early, i.e., by 24 h postchallenge. Likewise, a number of carbohydrate and amino acid fermentation products began to increase in abundance by 24 and 30 h, at which point we detected an increase in the abundances of free amino acids and dipeptides with concomitant increases in protease and peptidase gene expression. Future studies of the activity of C. difficile proteases and peptidases *in vivo* are needed to determine what role, if any, they play in nutrient acquisition and whether the tissue damage induced by the toxins is required to liberate potential energy sources. Additionally, by defining what is required for C. difficile physiology and pathogenesis *in vivo*, it will allow us to rationally design more highly targeted bacterial therapeutics to outcompete and prevent this infection in the future.

## MATERIALS AND METHODS

### Ethics statement.

Ethics and animal housing conditions were identical to those previously described by Theriot et al. (9, 12). Briefly, the University Committee on the Care and Use of Animals at the University of Michigan approved this study. The University of Michigan laboratory animal care policies follow the Public Health Service policy on Humane Care and Use of Laboratory Animals. Animals were assessed twice daily for physical condition and behavior, and those assessed as moribund were humanely euthanized by CO_2_ asphyxiation. Trained animal technicians performed animal husbandry in an AAALAC-accredited facility.

### Animals and housing.

C57BL/6 wild-type (WT) mice (5 to 8 weeks old; male and female) from a breeding colony that was established using animals purchased from Jackson Laboratories (Bar Harbor, ME) were used for the experimental infections. Mice were housed with autoclaved food, bedding, and water. Cage changes were performed in a laminar flow hood. Mice were subjected to a 12 h cycle of light and darkness.

### Mouse model of C. difficile infection.

C. difficile VPI 10463 (ATCC 43255) spores were prepared as described in previous studies (9, 12). Briefly, C. difficile spores were heat treated for 20 min at 65°C to ensure that any remaining vegetative bacilli were killed before animal gavage was performed. Viable spores were enumerated by plating for CFU per milliliter on taurocholate, cefoxitin, cycloserine, and fructose agar (TCCFA) to determine the challenge dose (81). Mice (*n* = 32; male and female) were given cefoperazone (0.5 mg/ml) in sterile drinking water for 5 days and were allowed 2 days on regular drinking water before challenge with 820 C. difficile spores was performed by oral gavage. Mice (*n* = 8; male and female) from different cages were euthanized by CO_2_ asphyxiation and subjected to necropsy prior to C. difficile challenge at time point 0 h and throughout the infection period at 12 h, 24 h, and 30 h. Cecal content was collected at the time of necropsy and stored in RNAlater for transcriptomic analysis and/or flash frozen in liquid nitrogen for metabolomic analysis. Samples were kept at −80°C until processing. Animals challenged with C. difficile were monitored for signs of clinically severe CDI, including inappetence, diarrhea, and hunching. At the time of necropsy, cecal content of animals challenged with C. difficile (*n* = 4) was plated on selective TCCFA to confirm colonization and enumerate bacterial load. All samples stored at −80°C in this study were later shipped on dry ice and stored at −80°C at C. M. Theriot’s new institution, North Carolina State University, until further processing.

### Global metabolomic analysis.

Cecal content was harvested from mice at 0 h (before C. difficile challenge) and at 12 h, 24 h, and 30 h postchallenge with C. difficile VPI 10463 spores (*n* = 8 per time point; 4 male and 4 female from different cages). Cecal content samples were submitted in 1.5-ml Eppendorf tubes to Metabolon, Inc. (Durham, NC), for untargeted metabolomics analysis. Sample preparation for metabolomics analysis was performed by Metabolon, Inc., in the same manner as was described in our previous study (9) and in the extended Methods section in Text S1 in the supplemental material. Briefly, individual samples were subjected to methanol extraction and then split into aliquots for analysis by ultra-high-performance liquid chromatography-mass spectrometry (UHPLC/MS). The global biochemical profiling analysis comprised four unique arms consisting of reverse-phase chromatography positive-ionization methods optimized for hydrophilic compounds (LC/MS Pos Polar) and hydrophobic compounds (LC/MS Pos Lipid) and reverse-phase chromatography performed under negative-ionization conditions (LC/MS Neg) as well as a hydrophilic interaction liquid chromatography (HILIC) method coupled to negative ionization (LC/MS Polar) (82). All the methods alternated between full-scan MS and data-dependent MS^*n*^ scans. The scan ranges differed slightly between methods but generally covered 70 to 1,000 *m/z*.

10.1128/mSphere.00089-18.1 TEXT S1  Extended Methods section for global metabolomics analysis. Download TEXT S1, DOCX file, 0.1 MB. Copyright © 2018 Fletcher et al. 2018 Fletcher et al. This content is distributed under the terms of the Creative Commons Attribution 4.0 International license.

Metabolites were identified by automated comparison of the ion features in the experimental samples to a reference library of chemical standard entries that included retention time, molecular weight (*m/z*), preferred adducts, and in-source fragments as well as associated MS spectra and were curated by visual inspection for quality control using software developed at Metabolon. Identification of known chemical entities was based on comparisons to metabolomic library entries of purified standards (83).

Two types of statistical analyses were performed: (i) significance tests and (ii) classification analyses. Standard statistical analyses were performed in ArrayStudio on log-transformed data. For those analyses that are not standard analyses available in ArrayStudio, R software (https://cran.r-project.org/) was used. Following log transformation and imputation of missing values, if any, with the minimum observed value for each compound, contrast ANOVA was used as a significance test to identify biochemicals that differed significantly (*P* < 0.05) among all time points. An estimate of the false-discovery-rate (*q*) value was calculated to take into account the multiple comparisons that normally occur in metabolomic-based studies. For the scaled-intensity graphics, each biochemical in the original scale (raw area count) was rescaled to set the median across all animals and time points to a value of 1.

Additional statistical analyses and data visualization was performed in MetaboAnalyst 3.0 (http://www.metaboanalyst.ca/faces/ModuleView.xhtml) (31). Briefly, the data were uploaded in the Statistical Analysis module with default settings and no further data filtering. The data were log transformed using the glog option, and the Kruskal-Wallis one-way ANOVA option was used to determine statistical significance. The heat map was built using the top 50 metabolites identified by Random Forest analysis with the Ward clustering algorithm and Euclidean distance.

### Extraction of C. difficile RNA from cecal content.

Paired samples of cecal content (*n* = 4 per time point; 2 males and 2 females at 12 h, 24 h, and 30 h) harvested for the untargeted metabolomics analyses were suspended in RNAlater (Thermo Fisher Scientific) and stored at −80°C until RNA extraction, at which point the samples were centrifuged and the RNAlater supernatant was removed. Pelleted cecal content was resuspended in 10 ml TRIzol reagent (Thermo Fisher Scientific) and distributed to 1.5-ml centrifuge tubes in 1-ml aliquots. Due to the volume of the tubes, the RNA extraction was performed with two samples at a time. Phase separation was performed using 200 µl chloroform per 1 ml cecal content/TRIzol. The aqueous phase (~500 µl) was added to ice-cold isopropanol with 5 µg/ml glycogen at 1:1. Samples were centrifuged at 4°C for 20 min, after which pellets were washed three times with 70% ethanol. Pellets were resuspended in water and stored at −80°C until further processing. RNA quality was assessed via the use of an Agilent 2100 Bioanalyzer. All subsequent manipulations were performed on all samples simultaneously. RNA samples were depleted of DNA by two rounds of treatment with Turbo DNase (Thermo Fisher) per the manufacturer’s protocol; all samples were column purified with a Zymo Clean and Concentrator kit (R1015). Depletion of contaminating genomic DNA was confirmed via PCR performed with *rpoC* primers (see Table S1 in the supplemental material).

### RNA Seq library preparation and analysis.

RNA was assessed for quality using a BioAnalyzer (Agilent Technologies, Santa Clara, CA). Samples with RNA integrity numbers (RINs) of 8 or greater were depleted of rRNA using RiboZero (Illumina catalog no. MRZH116). One of 12 samples was not used due to a poor RIN score (less than 8). The rRNA-depleted RNA was sent to the University of Michigan DNA Sequencing Core, Ann Arbor, MI, where samples were processed in a blind manner and converted to a library capable of cluster generation and sequencing using a TruSeq Stranded mRNA Library Prep kit (Illumina catalog no. RS-122-2001 and RS-122-2001) per the supplier’s protocol. Libraries were checked for size on a TapeStation and quantified using a Kapa Biosystems library quantification kit (catalog no. KK4835) for Illumina adapters. The libraries were pooled and sequenced on a HiSeq 4000 system as a paired-end 50-nucleotide run following the Illumina protocol. The input RNA (100 ng) underwent 12 cycles of PCR, and 11 libraries were multiplexed and run across 5 lanes to reduce lane-to-lane or run-to-run variation. Raw fastq.gz files were imported into Geneious 10.2 (Biomatters) (51). BBDuk was used in Geneious to trim adapters, low-quality bases (Phred score of less than 30), and short reads (less than 30 nucleotides), as well as reads with an average Phred score of less than 30. Trimmed paired-end reads were then mapped to the C. difficile VPI 10463 genome (NZ_CM000604) using the Geneious mapper with default settings, mapping only those reads in which each member of the pair mapped at the expected distance from the other member, resulting in averages of 9.74 × 10^4^, 1.49 × 10^6^, and 1.45 × 10^6^ reads mapping at 12, 24, and 30 h, respectively. Differential expression analysis was performed with the DESeq2 plugin within Geneious, defining a gene as differentially expressed if there was at least a 2-fold change in expression with an adjusted *P* value (*P*-adj.) of <0.05 to account for multiple-testing results (52). A Venn diagram of unique and shared differentially expressed genes was generated in Venny 2.1 (84), and volcano plots were constructed in R. Protein sequences from every differentially expressed gene were obtained via Batch Entrez and loaded into Blast2GO for Gene Ontology mapping, Enzyme Commission (EC) assignment, and mapping onto KEGG pathways. Among the 24 h versus 12 h DEGs, 237 of 297 successfully completed the Blast2GO pipeline and were assigned Gene Ontology (GO) annotations, as did 390 of 520 DEGs from the 30 h versus 12 h comparison; 12 of 14 from the 30 h versus 24 h comparison completed the pipeline. Many of those that did not complete the pipeline were proteins of unknown function for which no GO annotation could be assigned or those with no homologs identified via BLAST. These sequences were also uploaded to the STRING 10.5 database via the Web interface for prediction of protein interaction networks and enrichment analysis of KEGG pathways (85). No changes from the default settings were made. The predicted interaction networks for C. difficile VPI 10463 are unavailable in the STRING 10.5 database, so those for C. difficile 630 were used instead. Briefly, the STRING database compares the number of edges between the nodes in a network to those in a random network of proteins of similar number and performs Fisher’s exact test with multiple-comparison corrections to ascertain if pathways are enriched in the submitted network.

### Reverse transcription and quantitative real-time PCR.

DNA-depleted RNA was used as the template for reverse transcription performed with Moloney murine leukemia virus (MMLV) reverse transcriptase (NEB) following the manufacturer’s protocol. The cDNA samples were then diluted 1:5 in water and used in quantitative real-time PCR with gene-specific primers (Table S1) using SsoAdvanced Universal Sybr green Supermix (Bio-Rad) according to the manufacturer’s protocol. Amplifications were performed in technical triplicate, and copy numbers were calculated by the use of a standard curve and normalized to that of the housekeeping gene *rpoC*.

### Multivariate-based integration of the gut metabolome and C. difficile transcriptome.

To identify associations between the gut metabolome and C. difficile transcriptome, we performed a sparse partial least-squares-discriminant analysis (sPLS-DA) as implemented in the mixOmics package (86). sPLS-DA is a supervised approach that combines dimensionality reduction with variable selection through penalization (87). Within the mixOmics package, we applied the framework DIABLO, which focuses on the integration of multiple omics measurements across *n* samples. We used the 11 pairwise samples of the transcriptomics and metabolomics. Prior to utilizing the mixOmics package, we preprocessed the data and used only those variables where the measurements had a standard deviation of greater than 0.1 across all time points. Additionally, we calculated the median absolute deviation for the transcriptomics data and also utilized a threshold of 0.1 (88). Preprocessing the data reduced our variables to 621 metabolites and 1,771 transcripts across 11 time points as follows: three samples at the 12 h time point and four samples each at the 24 h and 30 h time points.

The aim of the analysis was to identify a highly correlated multiomics signature discriminating the time points throughout infection at 12 h, 24 h, and 30 h. We assumed that the transcriptome and metabolome data were highly correlated and choose a design matrix where all blocks are connected with a link value of 0.9. We tested this design link at values of 0.1 to 0.9 and did not find any noticeable differences for varied correlation links. We fitted an sPLS-DA model, assessed the global performance, and optimized the number of components. We choose the two-component approach on the basis of the decrease in the overall balanced error and of the overall error decrease of the centroid and maximum distances.

Using the optimal number of components, we then selected the optimum number of variables to use for sPLS-DA. We created a grid with values of 2 to 100 and used the leave-one-out cross-validation scores and the tuning function to determine the optimal sparsity parameters to classify the discrete outcome. The tuning process chooses 2 and 12 metabolites and chooses 34 and 6 transcripts on the first component and the second component, respectively, for the supervised analysis.

### Data availability.

Metabolomics data were deposited in the Metabolomics Workbench repository under study number ST000930. Raw sequences have been deposited in the Sequence Read Archive (SRA) with submission number SRP134023. The accession numbers are SAMN08639656, SAMN08639657, SAMN08639658, SAMN08639659, SAMN08639660, SAMN08639661, SAMN08639662, SAMN08639663, SAMN08639664, SAMN08639665, SAMN08639666. Other raw data are provided in the supplemental tables.
