# Supplementary material for: Shifts in the Gut Metabolome and Clostridium difficile Transcriptome throughout Colonization and Infection in a Mouse Model
Source: mSphere. 2018 Mar 28;3(2):e00089-18. doi: 10.1128/mSphere.00089-18 (PMC5874438; doi:10.1128/mSphere.00089-18)
Supplement: FIG S1 [file sph002182505sf1.pdf]

Carbohydrates

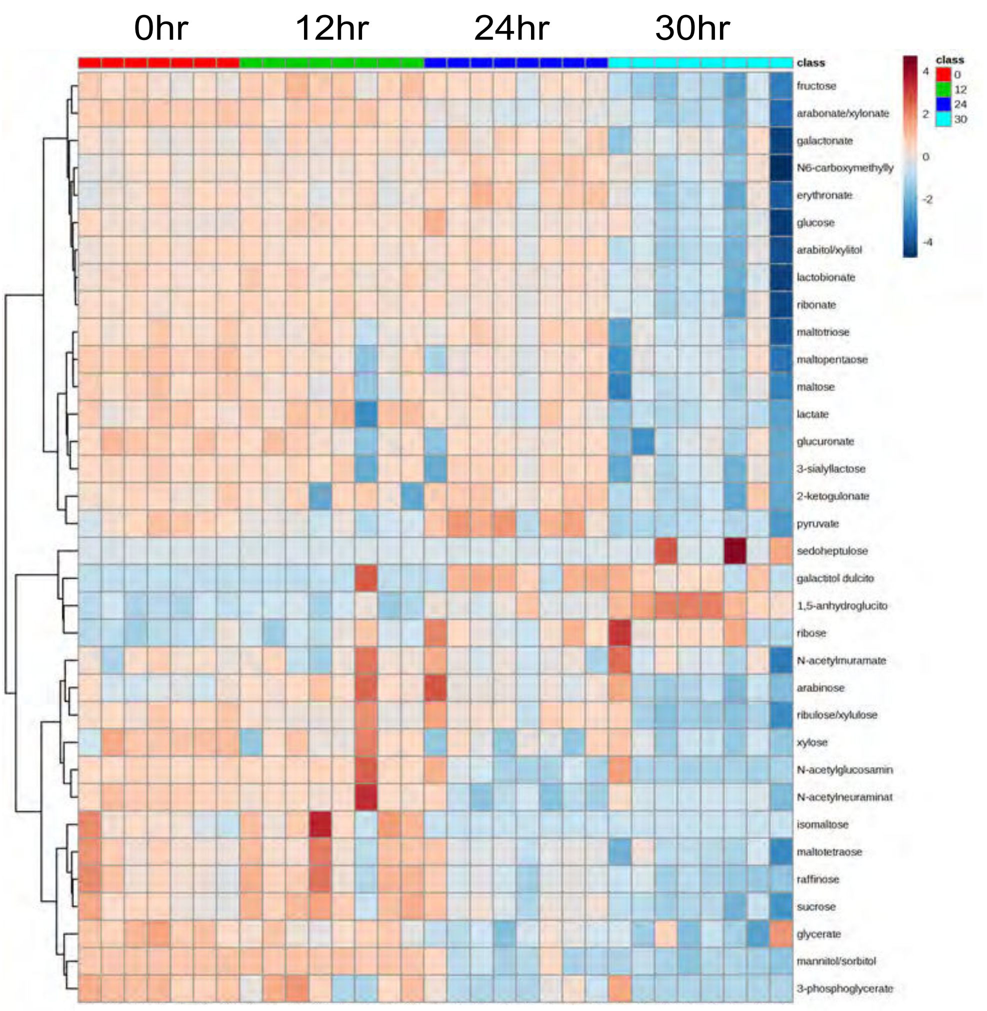

Peptides

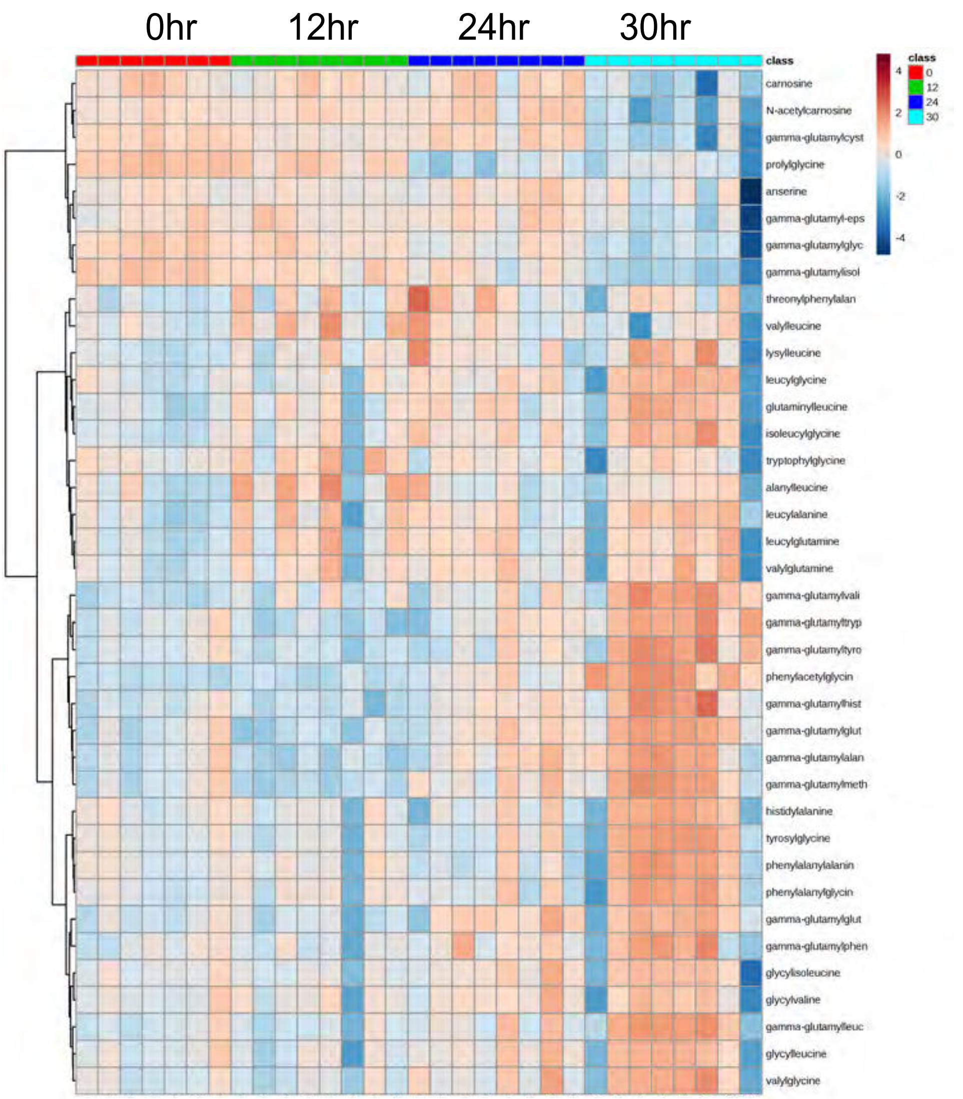

Figure S1. Heatmaps of the relative abundances of carbohydrates and peptides not included in the Random Forest in Figure 2. Each column corresponds to the cecal metabolome from an individual mouse, and each row corresponds to a given metabolite. Unsupervised hierarchical clustering was used to cluster metabolites with similar abundance profiles over time. The heatmap scales ranges from -4 to 4 on a $\log_2$ scale.
