# Supplementary figures and images for: Shifts in the Gut Metabolome and Clostridium difficile Transcriptome throughout Colonization and Infection in a Mouse Model
Source: mSphere. 2018 Mar 28;3(2):e00089-18. doi: 10.1128/mSphere.00089-18 (PMC5874438; doi:10.1128/mSphere.00089-18)

## Lipids

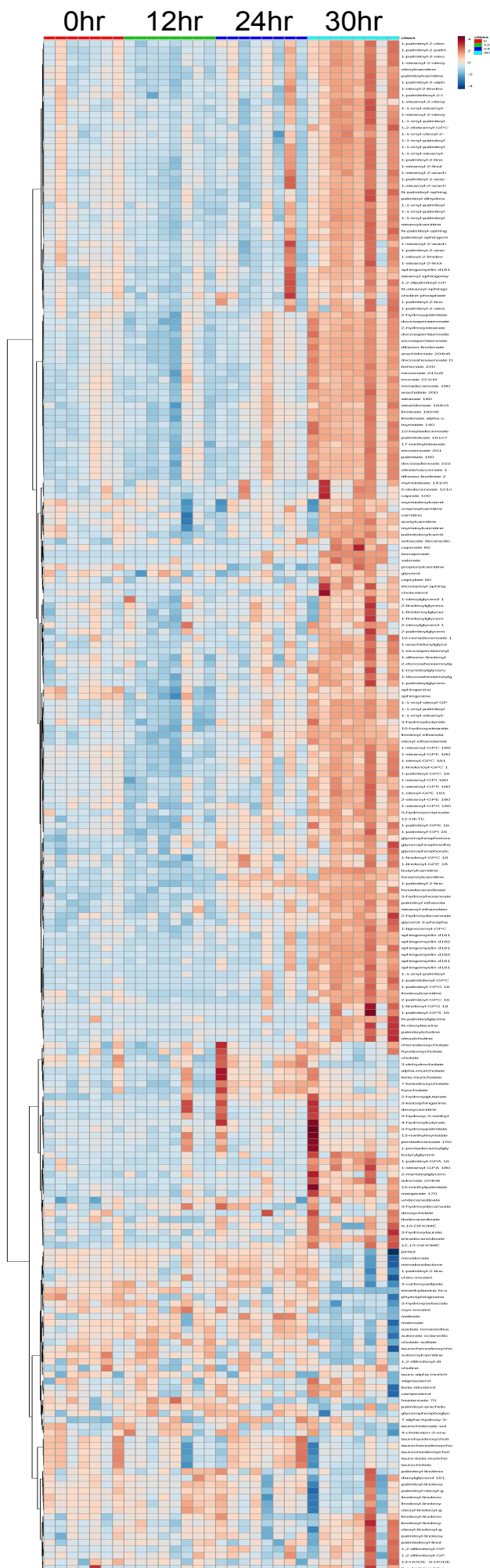

Supplement: FIG S2 [file sph002182505sf2.pdf]
