# Supplementary material for: Shifts in the Gut Metabolome and Clostridium difficile Transcriptome throughout Colonization and Infection in a Mouse Model
Source: mSphere. 2018 Mar 28;3(2):e00089-18. doi: 10.1128/mSphere.00089-18 (PMC5874438; doi:10.1128/mSphere.00089-18)
Supplement: FIG S3 [file sph002182505sf3.pdf]

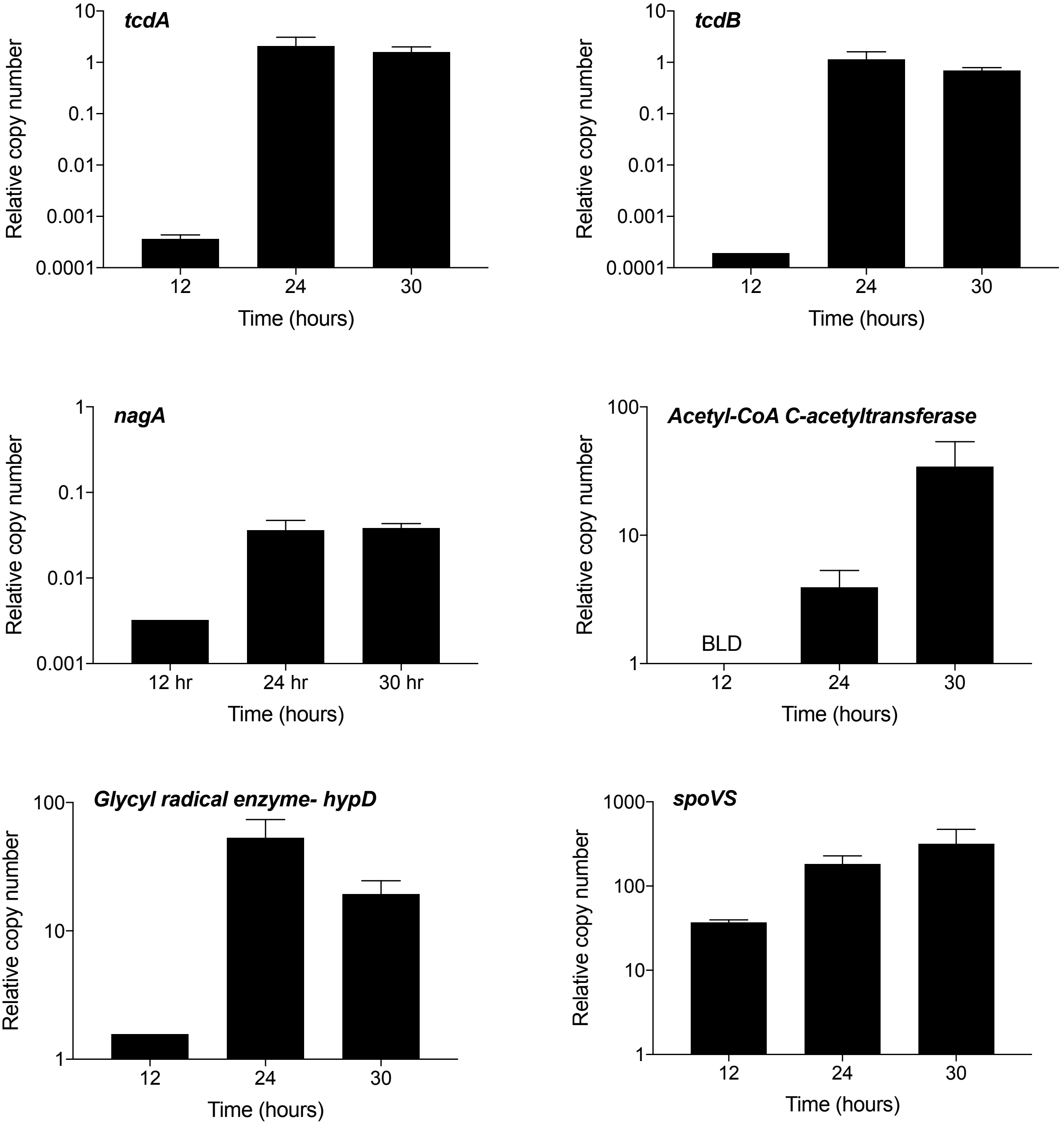

Figure S3. Quantitative reverse transcriptase PCR of six genes identified as differentially expressed in the RNA Seq analysis. Expression was quantified via standard curve and was normalized to the expression of *rpoC*. Information on primers can be found in Table S1.
