# Supplementary material for: Shifts in the Gut Metabolome and Clostridium difficile Transcriptome throughout Colonization and Infection in a Mouse Model
Source: mSphere. 2018 Mar 28;3(2):e00089-18. doi: 10.1128/mSphere.00089-18 (PMC5874438; doi:10.1128/mSphere.00089-18)
Supplement: TABLE S1 [file sph002182505st1.docx]

**Table S1.** Primers used in quantitative reverse transcriptase PCR in Fig. S4.

| Gene | Primer pairs |
| --- | --- |
| *rpoC* | 5’ – TGGCAGTCCATGTACCTTTATC  3’ – GGTGAACCATCTTTAGGAGCA |
| *tcdA* | 5’ – ACTAGACGAACATGACCCATTAC  3’ – GCTACCGTTGCAGCTATAGATAA |
| *tcdB* | 5’ – GGCAGCTGCTTCTGACATATTA  3’ – GGTCTGGTTGTATTCCTGGTAAC |
| *nagA* | 5’ - AGCCAGGTACAAAGGTATTATCA  3’ – AGGAAGGTCATCAGCAAGTC |
| *acetyl-CoA C-acetyltransferase* | 5’ – GTCCAATCCCTGCAACTAGAA  3’ – CTAATGCTTGGGCAGCAAAC |
| *glycyl radical enzyme* | 5’ – GCAAGACAAATGGCAGAAGAAG  3’ – CTGGTTTGTGAGCTGGTACA |
| *spoVS* | 5’ – GCGGGAATGCAAACTAAATCC  3’ - ACGAGGCGTTGCAGAAATA |
