# Supplementary material for: Shifts in the Gut Metabolome and Clostridium difficile Transcriptome throughout Colonization and Infection in a Mouse Model
Source: mSphere. 2018 Mar 28;3(2):e00089-18. doi: 10.1128/mSphere.00089-18 (PMC5874438; doi:10.1128/mSphere.00089-18)
Supplement: FIG S4 [file sph002182505sf4.pdf]

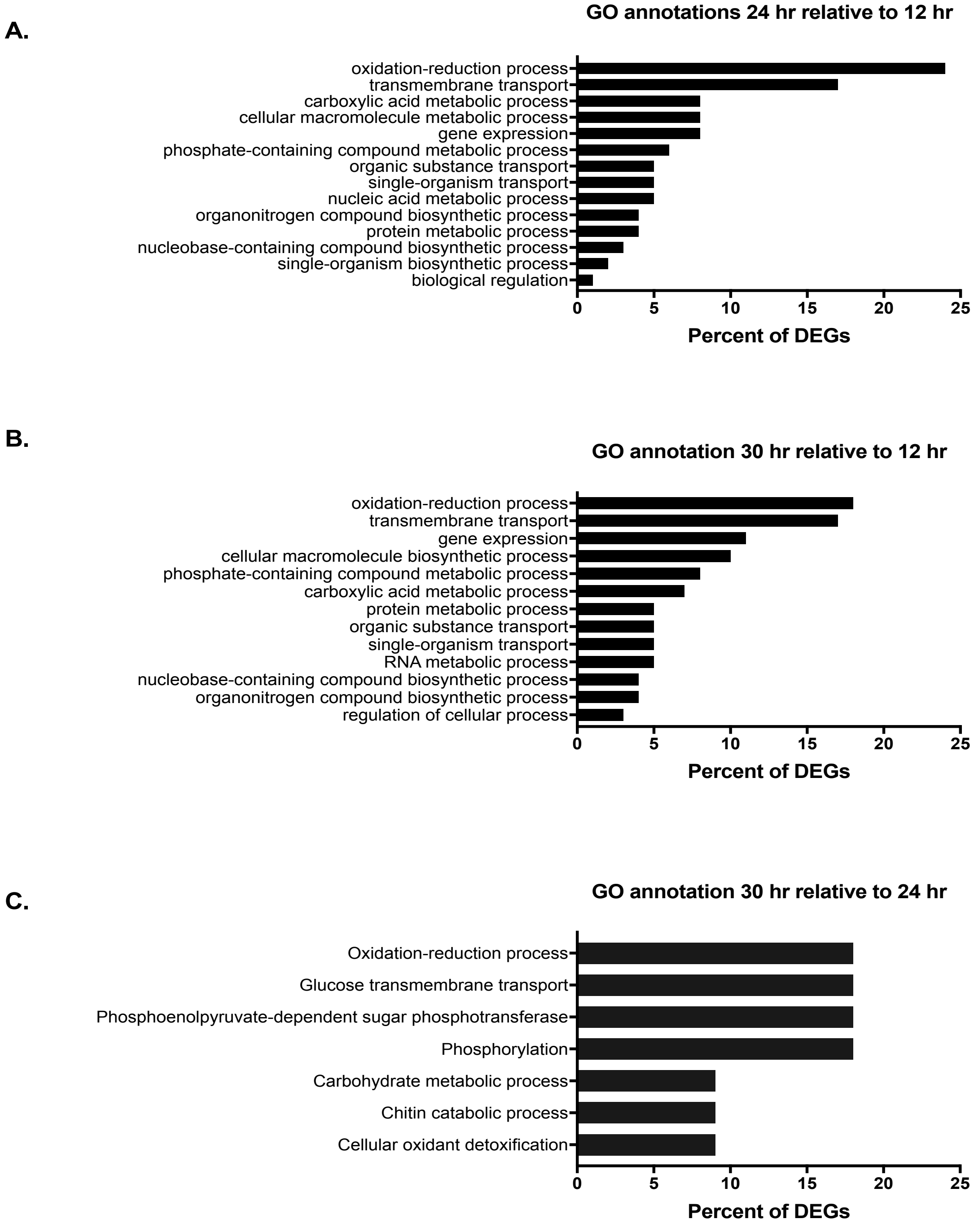

Figure S4. Blast2GO Gene Ontology annotations for the differentially expressed genes from each time point comparison during colonization and infection.
